# Information Resources Among Flemish Pregnant Women: Cross-sectional Study

**DOI:** 10.2196/37866

**Published:** 2022-10-11

**Authors:** Dorien Lanssens, Inge M Thijs, Pauline Dreesen, Ann Van Hecke, Pascal Coorevits, Gitte Gaethofs, Joyce Derycke, Inge Tency

**Affiliations:** 1 Faculty of Medicine and Life Sciences Limburg Clinical Research Center University of Hasselt Hasselt Belgium; 2 Obstetric Department and Future Health Limburg Clinical Research Center Ziekenhuis Oost-Limburg Genk Belgium; 3 Department Public Health and Primary Care Faculty of Medicine and Health Sciences Ghent University Gent Belgium; 4 Nursing Department Ghent University Hospital Gent Belgium; 5 Department of Midwifery Odisee University of Applied Sciences Sint Niklaas Belgium

**Keywords:** pregnancy app, mobile app, questionnaire, pregnancy, pregnant, mHealth, mobile health, cross-sectional, user need, user expectation, survey, maternal, maternity, user experience

## Abstract

**Background:**

There has been an exponential growth in the availability of apps, resulting in increased use of pregnancy apps. However, information on resources and use of apps among pregnant women is relatively limited.

**Objective:**

The aim of this study is to map the current information resources and the use of pregnancy apps among pregnant women in Flanders.

**Methods:**

A cross-sectional study was conducted, using a semistructured survey (April-June 2019) consisting of four different domains: (1) demographics; (2) use of devices; (3) sources of information; and (4) use of pregnancy apps. Women were recruited by social media, flyers, and paper questionnaires at prenatal consultations. Statistical analysis was mainly focused on descriptive statistics. Differences in continuous and categorical variables were tested using independent Student *t* tests and chi-square tests. Correlations were investigated between maternal characteristics and the women’s responses.

**Results:**

In total, 311 women completed the entire questionnaire. Obstetricians were the primary source of information (268/311, 86.2%) for pregnant women, followed by websites/internet (267/311, 85.9%) and apps (233/311, 74.9%). The information that was most searched for was information about the development of the baby (275/311, 88.5%), discomfort/complaints (251/311, 80.7%) and health during pregnancy (248/311, 79.7%), administrative/practical issues (233/311, 74.9%), and breastfeeding (176/311, 56.6%). About half of the women (172/311, 55.3%) downloaded a pregnancy app, and primarily searched app stores (133/311, 43.0%). Pregnant women who are single asked their mothers (22/30, 73.3%) or other family members (13/30, 43.3%) for significantly more information than did married women (mother [in law]: 82/160, 51.3%, *P*=.02; family members: 35/160, 21.9%, *P*=.01). Pregnant women with lower education were significantly more likely to have a PC or laptop than those with higher education (72/73, 98.6% vs 203/237, 85.5%; *P*=.008), and to consult other family members for pregnancy information (30/73, 41.1% vs 55/237, 23.1%; *P*<.001), but were less likely to consult a gynecologist (70/73, 95.9% vs 198/237, 83.5%; *P*=.001). They also followed more prenatal sessions (59/73, 80.8% vs 77/237, 32.5%; *P*=.04) and were more likely to search for information regarding discomfort/complaints during pregnancy (65/73, 89% vs 188/237, 79.5%; *P*=.02). Compared to multigravida, primigravida were more likely to solicit advice about their pregnancy from other women in their social networks (family members: primigravida 44/109, 40.4% vs multigravida 40/199, 20.1%; *P*<.001; other pregnant women: primigravida 58/109, 53.2% vs multigravida 80/199, 40.2%; *P*<.03).

**Conclusions:**

Health care professionals need to be aware that apps are important and are a growing source of information for pregnant women. Concerns rise about the quality and safety of those apps, as only a limited number of apps are subjected to an external quality check. Therefore, it is important that health care providers refer to high-quality digital resources and take the opportunity to discuss digital information with pregnant women.

## Introduction

Access to reliable information is critical to women’s experiences and well-being during pregnancy and childbirth [1,2]. Information and education help women understand what is happening and what can happen during their pregnancy [3] and can improve women’s satisfaction with the childbirth experience [4]. Pregnant women seek information to feel more confident and comfortable in their communication with health care providers, to make decisions during the perinatal period, and to prepare themselves for their maternal responsibilities [4-8]. Fulfilling a woman’s information needs depends on her access to adequate resources and her ability to comprehend what has been presented to her [7]. In the current context of our information-rich society, women are exposed to a wide range of information sources. This includes information sources from the health care system, conventional sources (eg, family, peers, and books), and digital information sources (eg, websites, apps, and social media) or eHealth [9,10].

The term eHealth refers to the application of electronic information and communication techniques in health care, primarily intended to improve patients’ health and the quality of care. eHealth is one of the fastest growing domains within health care [11,12]. Mobile health (mHealth) is a component of eHealth and is defined as the use of mobile technologies such as smartphones, tablets, computers, and other wireless devices (eg, pedometers, smartwatches) to support health services and improve the quality and efficiency of care [11,13-15].

mHealth is on the rise in health care, resulting in the exponential growth of mHealth apps [12,14,16]. The largest group of smartphone users are Millennials, those currently aged 18-34 years, which aligns with the time when many first experience pregnancy and parenthood [17,18]. Therefore, it is not surprising that there is a wide range of mobile apps on the topics of pregnancy, birth, and parenthood, with more than 1000 pregnancy apps available in the various app stores [19-23]. In line with the general trend in mHealth apps, an increase in the use of pregnancy apps has been observed [21,24,25]. The vast majority of pregnant women download on average 3 apps during pregnancy [24], and nearly one-quarter use these apps almost daily [22]. However, health care professionals are concerned about the quality, validity, and accuracy of information freely available through mobile apps [22,24-30] and the reliability and safety of these apps [22,24,25,27,28]. Further, the women may not be able to determine the accuracy of the information, as well as the accessibility and readability of online resources [29]. Research has showed a low level of concern about the validity of the information in the pregnancy apps, since 74% of users did not check the sources of information [22]. Incorrect and contradictory information can introduce unnecessary confusion, worries, anxiety, and uncertainty among pregnant women [29,31].

Considering the widespread use of the internet and smartphones as means to access health care information and as tools for health care management, it is interesting to know why and how pregnant women use online tools and what kind of information and features they are looking for [21]. However, the actual usage patterns and characteristics of women using pregnancy apps are relatively unknown [30]. In addition, information on information sources and the experience of women in Flanders (the northern Dutch-speaking region of Belgium) using digital tools during pregnancy is lacking. Therefore, the objective of this study was to map the current information resources of pregnancy apps and the use of pregnancy apps among pregnant women in Flanders.

## Methods

### Study Design and Population

A cross-sectional study was conducted, using a semistructured questionnaire that was distributed in Flanders from April 2019 to June 2019. This questionnaire was developed by researchers of Odisee University of Applied Sciences (Sint-Niklaas) and the Limburg Clinical Research Center/Mobile Health Unit (University of Hasselt – ZOL), based on literature and pre-existing questionnaires. Four different domains were questioned: (1) demographics of the pregnant women; (2) use of devices; (3) sources of information; and (4) use of pregnancy apps. A convenience sampling method was used to collect the data. Pregnant women were recruited through two different methods. The first method was the use of flyers in the waiting room of prenatal consultations. If pregnant women were interested in participating in this study, they received a paper questionnaire that they could fill in and return to the midwife at the prenatal consultation. The other method used was an online call for participation on the social media accounts of the participating hospital and universities. This online flyer contained a web-based link to an online survey. The same questions were asked in the online and paper questionnaires.

Pregnant women were recruited by researchers of the Limburg Clinical Research Center/Mobile Health Unit (group 1) and Odisee University of Applied Sciences (group 2). Data from group 1 was received via the prenatal ward of the Ziekenhuis Oost-Limburg (ZOL, Genk, Belgium), a tertiary hospital in Limburg. The Limburg Clinical Research Center/Mobile Health Unit is a part of the University of Hasselt. Data from group 2 were received via the prenatal ward of VITAZ (Sint-Niklaas, Belgium), a secondary hospital in East Flanders. The data collection at this hospital was performed by Odisee University.

### Data Exclusion

A total of 331 answers were received (group 1: n=268, 81%; group 2: n=63, 19%), of which 20 (6%) were not completely filled in (all from group 1). Therefore, 311 questionnaires (94%) were analyzed, of which 92.5% were from group 1 and 7.5% from group 2. Responses were compared online to verify and possibly exclude duplicates. No duplicates were retained.

### Statistical Analysis

Statistical analyses were performed with SPSS (version 22.0; IBM Corp). The statistical analyses were mainly focused on descriptive statistics (frequencies, percentages). Normality was tested by the Shapiro-Wilk test. Differences in continuous and categorical variables were tested using independent Student t tests and chi-square tests, respectively. Correlations were investigated between the characteristics of the pregnant women (marital status, educational level, occupation, gravidity) and their responses. All statistical analyses were done at nominal level *P*=.05.

### Ethical Considerations

The study was approved by the Medical Ethics Committees of the hospital (Ziekenhuis) Oost-Limburg (ZOL; Genk; no. 19/0026U, eudract/B-no. B371201939699) and Ghent University Hospital (EC 2018/0120, B-no. B670201835156). The survey was anonymous. An information letter was added to the survey to explain the context of the study. By completing the questionnaires, the participants automatically agreed to the terms of the study.

## Results

### Participant Demographics

In total, 311 questionnaires were completed and returned (group 1: 248/311, 79.7% vs group 2: 63/311, 20.3%). There were no significant differences in characteristics between the two groups, except for education level; group 2 had a significantly higher prevalence of participants with a high school and/or university education compared to group 1 (92.1% vs 72.2%; *P*=.004). In both groups, the mean age of the women was 30 years. Most of the women were married or in a civil partnership with their partner, were employees, and multigravida. The details of the characteristics are presented in Table 1. The difference in educational level did not influence the results of this study, so the results of the total study population will be discussed (and will not be divided by group).

**Table 1:** Characteristics of the women.

			Group 1 (n=248)		Group 2 (n=63)		*P* value (2-tailed)			Total (group 1 + group 2), N=311	
Age (years), mean (SD)			30.76 (4.08)		30.13 (4.06)		.27			30.63 (4.08)	
**Marital status, n (%)**								.84			
	Married	131 (52.8)		31 (49.2)					162 (52.1)		
	Living together	94 (37.9)		25 (39.7)					119 (38.3)		
	Single	23 (9.3)		7 (11.1)					30 (9.6)		
**Educational level, n (%)**								.004			
	Lower secondary school and/or higher secondary school	68 (27.4)		5 (7.9)					73 (23.5)		
	High school and/or university	179 (72.2)		58 (92.1)					237 (72.6)		
**Occupation, n (%)**								.40			
	Self-employed	17 (6.9)		7 (11.1)					24 (7.7)		
	Employee	185 (74.6)		49 (77.8)					234 (75.2)		
	Worker	21 (8.5)		3 (4.8)					24 (7.7)		
	Housewife	9 (3.6)		0 (0)					9 (2.9)		
	Unemployed	5 (2)		2 (3.2)					7 (2.3)		
	Student	5 (2)		0 (0)					5 (1.6)		
	Other	6 (2.4)		1 (1.6)					7 (2.3)		
Primigravida, n (%)			82 (33.1)		28 (44.4)		.09			110 (35.4)	

### Use of Electronic Devices

In this manuscript, a computer/laptop is defined as “an electronic device for storing and processing data, typically in binary form, according to instructions given to it in a variable program,” while a mobile phone is defined as “a telephone with access to a cellular radio system so it can be used over a wide area, without a physical connection to a network.” A smartphone/iPhone is defined as “a mobile phone that performs many of the functions of a computer, typically having a touchscreen interface, internet access, and an operating system capable of running downloaded apps,” and a tablet PC/iPad/iPod is defined as “a wireless touch screen personal computer (PC) that is smaller than a notebook but larger than a smartphone… modern tablets are built with wireless Internet or local area networks (LAN) and a variety of software applications, including business applications, Web browsers and games.”

The first domain of the questionnaires was about device use as well as the frequency of use (Table 2). The majority of devices were used daily by the women (computer/laptop: 127/311, 40.8%; mobile phone: 251/311, 80.7%; and smartphone/iPhone: 303/311, 97.4%).

**Table 2:** The use of devices and the frequency of use (n=311).

Device, n (%)	Every day	Several times per week	Several times per month	Once per month	Less than once per month	Never
Computer/laptop	127 (40.8)	83 (26.7)	39 (12.6)	21 (6.8)	29 (9.3)	6 (1.9)
Mobile phone	251 (80.7)	2 (0.6)	0 (0)	0 (0)	1 (0.3)	46 (14.8)
Smartphone/iPhone	303 (97.4)	3 (1)	1 (0.3)	0 (0)	0 (0)	1 (0.3)
Tablet PC/iPad/iPod	61 (19.6)	60 (19.3)	34 (10.9)	13 (4.2)	48 (15.4)	78 (25)

### Sources of Information

Of the 311 respondents, 267 women (85.9%) reported that they searched online for information about pregnancy. Table 3 gives an overview of the manner in which they gathered information about pregnancy. The obstetrician was their first source of information (268/311, 86.2%), followed by websites/the internet (267/311, 85.9%), and apps (233/311, 74.9%). The midwife was in fifth place (284/311, 59.3%), after friends (194/311, 62.4%).

The information that they sought was mostly about the following themes: development of the baby (276/311, 88.5%), discomfort/complaints during pregnancy (251/311, 80.7%), health during pregnancy (248/311, 79.7%), administration and practical matters (233/311, 74.9%), and breastfeeding (176/311, 56.6%).

**Table 3:** Sources of information accessed by participants (n=311).

Source of information	Participants, n (%)
Obstetricians	268 (86.2)
Websites/the internet	267 (85.9)
Apps	233 (74.9)
Friends	194 (62.4)
Midwife	184 (59.2)
Mother (in law)	179 (57.4)
Media	172 (55.3)
Social media	155 (49.8)
General practitioner	142 (45.7)
Other pregnant women	138 (44.4)
Books	131 (42.1)
Infosessions for future parents	111 (35.7)
Partner	103 (33.1)
Child and family	100 (32.2)
Other family members	84 (27)
Sisters	76 (24.4)
No information searched	2 (0.6)

### Use of Pregnancy Apps

Of the 311 women, 55.3% downloaded a pregnancy app (172/311). The mean number of downloaded apps was 1.59 (SD 0.96), with a maximum number of 7 apps. The top 3 reasons for downloading a pregnancy app were (1) to have a calendar to follow the growth and development of the baby (104/149, 69.8%); (2) to receive push messages with information, advice, and tips about the pregnancy on a daily/weekly basis (103/149, 69.1%); and (3) to have checklists (for baby names, baby layettes, etc; 38/189, 25.5%). The top 5 ways that women were informed about the existence of the apps were (1) a search in app stores (74/172, 43.0%); (2) friends (13/172, 7.6%); (3) a combination of a search in app stores and (social) media (9/172, 5.2%); (4) a combination of a search in app stores and friends (7/172, 4.1%); and (5) social media (7/172, 4.1%). Advice from a health care provider (midwife) was mentioned by only one respondent (1/172, 0.6%). Women downloaded the apps because they were provided by a reliable organization or institution (10/172, 5.8%) or because of the good ratings by other users (10/172, 5.8%). Only 1.7% (3/172) of the women used an app on the advice of their health care provider. The downloaded app was visited daily by 9.3% (16/172) of the women, weekly by 9.3% (16/172), and monthly by 2.9% (5/172). Few women (5/172, 2.9%) reported rare app use. Only 3.5% (6/172) of the women paid for an app (€2-€5, US $2.04-$5.10). The most frequently downloaded app was the Pregnancy + app (76/172, 44.2%), which is a worldwide pregnancy app developed by Philips.

### Correlations With Maternal Factors

Differences between maternal characteristics (marital status, education, and gravidity) and use of devices, source of information, and use of pregnancy apps were investigated. Only significant results will be discussed below. A detailed overview of all results is provided in Multimedia Appendices 1-3.

#### Marital Status

There is a significant association between the source of information and a woman’s marital status: single women were significantly more likely to ask their mothers (73.3%) or other family members (43.3%) for information than were married pregnant women (mother [in law]: 51.3%; *P*=.02).

#### Education

Pregnant women with a lower education level (lower secondary school and/or higher secondary school) were significantly more likely to use a PC or laptop in their occupation than pregnant women who had a higher education level (high school and/or university; 98.6% vs 85.5%; *P*<.01). They also were more likely to consult other family members for information about their pregnancy (41.1% vs 23.1%; *P*<.01) and less likely to consult their gynecologist (95.9% vs 83.5%; *P*=.001). In addition, they followed more prenatal sessions (80.8% vs 32.5%; *P*=.04) and searched more for specific information about their discomfort and complaints during pregnancy (89% vs 79.5%; *P*=.02).

#### Gravidity

Compared to multigravida, primigravida were more likely to ask for advice about their pregnancy from those in their vicinity, such as family members (primigravida: 40.4% vs multigravida: 20.1%; *P*<.001) and other pregnant women (primigravida: 53.2% vs multigravida 40.2%; *P*=.03). Primigravida also searched for more specific information about their pregnancy compared to multigravida. The specific significant results are general health (primigravida: 64.2% vs multigravida: 41.2%; *P*<.001), health during pregnancy (primigravida: 89% vs multigravida: 75.9%; *P*=.01), sexuality (primigravida 37.6% vs multigravida 10.6%; *P*<.001), emotions, experiences and mood (primigravida: 37.6% vs multigravida: 25.1%; *P*=.02), and administrative and practical matters (primigravida: 82.6% vs multigravida: 71.9%; *P*=.04).

## Discussion

### Principal Results and Comparison With Prior Work

There is relatively little known about the sources of information in pregnancy apps and actual use of these apps by pregnant women, including their characteristics. To our knowledge, this is the first study that sought to map the current information resources and use of websites and mobile apps of Flemish women and their needs and expectations regarding digital information.

The use of websites and mHealth is becoming an increasingly important way for women to receive information about their pregnancy [21,24,25]. Our study showed that most devices (computer/laptop, mobile phone, and smartphone/iPhone) were used daily by women. More than half of the women downloaded a pregnancy app (55.3%), with an average of 1 or 2 apps per pregnancy. This is in line with a study by Lee et al [24], who reported that the average number of free apps downloaded was 2.4 (SD 1.57) [24].

The 3 most common reasons that women in our study downloaded an app were (1) follow-up of the growth and development of the baby (69.8%), (2) daily/weekly notifications based on push messages with information, tips, and advice about the pregnancy (69.1%), and (3) use of checklists (25.5%). A possible reason for this is that this kind of information is easy to explain and very accessible for everyone. In addition, women’s care providers (eg, obstetrician, midwife) are not available at every moment of the day, but an app is. In studies by Wang et al [23] and Lupton and Pederson [22], the most reported reason for app usage was for monitoring fetal development (81.5% and 86%, respectively). In addition, the need for tailored advice and tracking of pregnancy changes, enabled by notifications, is another important reason for app usage. Further research is needed to gain insight into reasons for app usage, how app functions meet the expectations of women during pregnancy, and to what extent apps can be complementary to and integrated into current prenatal care.

The majority of women in our study (85.9%) searched for information on the internet, particularly information regarding fetal development, health and complaints during pregnancy, and practical and financial issues. This is in line with other studies showing that 65% [20] and 97% [32] of women sought digital health information. We also found that the obstetrician was the main source of information for many women (86.2%), followed by websites/internet (85.9%) and apps (74.9%). The midwife was in fifth place (59.3%). Our results seem to support those of previous studies [23,29,31,33,34], in which it was found that digital resources are a major source of information during pregnancy and childbirth, in combination with information and support from family, friends, and health professionals. We assume that the underlying reason for this is that online searches of apps are available at any time and place, and they are also accessible for questions that pregnant women might not want to ask their care providers about (eg, sensitive questions about finances).

Health care professionals are concerned about the quality, validity, and accuracy of digital information resources [22,24,25,27,28]. Previous research showed that women did not discuss information they found online with their midwife or doctor and between 8%-12% of women were unsure, worried, or confused about this information [29]. Findings were similar in a study by Wang et al [23] where women expressed the need for information about future apps from health care providers as well as the need to discuss contradictory information with health care providers. However, in our study, only 1.7% (3/172) of the women used an app on the advice of their health care provider, which is a lower percentage compared to a study by Mackintosh et al [29], where 30% of the women used websites or apps recommended by their midwife or doctor [29]. It seems that the recommendation of apps is not yet naturalized in the Flemish field of obstetrics, but this also indicates how important it is that health care providers refer women to high-quality digital resources such as websites and apps and take the opportunity to discuss digital information during consultations.

Similar to Buchanan et al (2021) [30] and Vogels-Broeke et al (2022) [34], our study also highlighted the importance of interpersonal resources such as peers, friends, and family for health- and parenting-related information. However, dependency on information from the internet and relatives can be problematic, particularly when this advice conflicts with recommendations from health professionals. Therefore, it may be recommended to disseminate digital health resources such as mobile apps to the social networks and family members of pregnant women to increase the likelihood of positive health outcomes [30].

Further, our study showed differences in sources of information depending on marital status, educational level, and gravidity. Single women were significantly more likely to ask their relatives for information (ie, their mother or other family members) than were married or cohabiting pregnant women, probably due to the fact that they do not have a partner to turn to with their questions and concerns. Pregnant women with a lower education level were significantly more likely to have a PC or laptop than those with higher education; they also were more likely to consult other family members for information about their pregnancy, followed more prenatal sessions, and searched more for online information on discomfort and complaints during pregnancy. This is in contrast with the results of Buchanan et al (2021) [30], who found that women from a lower socioeconomic background had lower rates of pregnancy app uptake, and were less likely to use written or online resources and digital technologies to search for health information [30]. A study by Vogel-Broeke et al [34] found that the use of websites was lower among women who had a low level of education versus those with a middle or high level of education. However, education level appeared to play little part in the online practices of women in the study by Mackintosh et al [29].

Finally, our study showed that primigravida asked for more advice about their pregnancy from those in their families and social circles compared with multigravida. Primigravida consulted their family members or other pregnant women and also searched for more specific information regarding general health information, health during pregnancy, sexuality, emotions and mental well-being, and administrative and practical issues. This could be because multigravida have already experienced a pregnancy, and learned a lot from that experience. The need for advice and information is lower compared to primipara. These findings are consistent with other literature indicating that women have the greatest need for information during their first pregnancy and are more likely to use health apps as a source of information [30,31,34]. This emphasizes the importance of customized information, adjusted to the needs of pregnant women. Maternal characteristics need to be considered when developing mobile apps to ensure uptake among pregnant women from broader sociodemographic backgrounds [30].

### Strengths and Limitations

To our knowledge, this is the first study that sought to map the current information resources and use of websites and mobile apps of Flemish women and their needs and expectations regarding digital information. Another strength of this study is that the survey was developed based on existing questionnaires and prior, similar investigations among postnatal women. However, the psychometric properties of the questionnaire were not determined.

This study has several limitations that need consideration. Given the small sample size and the fact that pregnant women were recruited in only two Flemish regions (Genk and Sint-Niklaas), findings may not be generalizable to all Belgian women. Second, there were two versions of the questionnaire: a web-based questionnaire and a paper-based questionnaire. We mainly recruited women through social media, which means that we probably reached women who are more digitally skilled and therefore more familiar with digital technologies. It is known that digital (health) literacy is limited among vulnerable people. A recent report on digital inclusion found that 32% of Belgians have weak digital skills. This value increases up to 75% for people with a low income and low educational level [35]. It is likely that vulnerable pregnant women are underrepresented in our study. We tried to overcome this issue by using paper-based questionnaires, but we are aware of the fact that this could lead to bias in the answers (eg, limited time on the prenatal ward, less privacy). Further, we measured the use of pregnancy apps at one time point, regardless of gestational age. It has been demonstrated that sources of information vary over the course of pregnancy and app use declines as pregnancy progresses [23]. Vogels-Broeke et al [34] also found differences in the use of (digital) information resources between early and late pregnancy. Therefore, a longitudinal approach to studying the use of digital resources among pregnant women is recommended. Finally, we only investigated the needs, expectations, and app usage of pregnant women; it would be interesting to investigate the attitudes and experiences of partners as well as health care professionals.

### Recommendations for Further Research

Health care professionals must be aware that women search for pregnancy information online. Further research is needed to establish how health care professionals can support women’s digital use during pregnancy and how digital information resources can be integrated into daily practice. Studies on health care professionals’ attitudes, needs, feasibility, and acceptability toward digital resources are recommended, as well as studies investigating how they are engaging and dealing with pregnancy and parenting apps in clinical practice. In addition, it would be interesting to investigate the experiences and actual app usage of partners. Further insights are needed on how pregnant women select apps, use them through the different trimesters, and evaluate their quality and usefulness.

### Conclusions

Health care professionals need to be aware that mobile health apps and the internet are important and growing sources of information for pregnant women—as shown in our study, they are the second and third most common sources of information. It is likely that this digital trend will continue in the future and will become even more important. Concerns arise about the quality and safety of such apps, as only a limited number of apps are subjected to an external quality check. Therefore, it is important that health care professionals refer patients to high-quality digital resources and take the opportunity to discuss digital information with pregnant women. The availability of high-quality, evidence-based, and customized mobile pregnancy apps represents an important opportunity to optimize maternal and birth outcomes. Efforts should be made by health care professionals, app developers, and policy makers to ensure the quality of health apps and their integration into maternal care.

## Appendix

Multimedia Appendix 1 Correlation between marital status (married vs single) and use of technology, information resources and topics, and use of pregnancy apps.

Multimedia Appendix 2 Correlation between educational level (secondary school vs higher education) and use of technology, information resources and topics, and use of pregnancy apps.

Multimedia Appendix 3 Correlation between gravidity (primigravida vs multigravida) and use of technology, information resources and topics, and use of pregnancy apps.
