# Supplementary material for: Information Resources Among Flemish Pregnant Women: Cross-sectional Study
Source: JMIR Form Res. 2022 Oct 11;6(10):e37866. doi: 10.2196/37866 (PMC9597425; doi:10.2196/37866)
Supplement: Multimedia Appendix 2 [file formative_v6i10e37866_app2.docx]

| **Table IV : Correlation between educational level (secondary school vs. higher education) and use of technology, information resources and topics and use of pregnancy apps** | | | |
| --- | --- | --- | --- |
| **Variables** | **Secondary school (or lower)**  **(n = 73)**  **(n, %)** | **Higher education**  **(n = 237)**  **(n,%)** | ***P* - value**  **(two-tailed)** |
| **USE OF TECHNOLOGY** |  |  |  |
| Do you have a computer or laptop? - Yes | 72 (98.6) | 203 (85.5) | **<.01** |
| Do you have Internet access? - Yes | 73 (100) | 232 (97.9) | .73 |
| Do you have a smartphone/iPhone? - Yes | 73 (100) | 234 (98.7) | NA |
| Do you have a tablet CP/iPAD/iPOD? - Yes | 53 (72.6) | 177 (74.67) | .74 |
| **INFORMATION RESOURCES** |  |  |  |
| I didn’t search for any information | 0 (0) | 2 (0.8) | .77 |
| Books | 34 (46.6) | 97 (40.9) | .48 |
| Media (magazine, newspapers, radio, television, etc.) | 42 (57.5) | 131 (55.3) | .50 |
| Websites/Internet | 68 (93.2) | 198 (83.5) | .11 |
| Social media (e.g. Facebook, etc.) | 29 (39.7) | 126 (53.2) | .08 |
| Mobile applications | 60 (82.2) | 173 (73) | .07 |
| Partner | 19 (26) | 37 15.6) | .12 |
| Mother (in law) | 44 (60.3) | 119 (50.2) | .19 |
| Sister(s) | 21 (28.8) | 55 (23.2) | .53 |
| Friend(s) | 53 (72.6) | 141 (59.5) | .06 |
| Other family members | 30 (41.1) | 55 (23.1) | **<.01** |
| Other pregnant women | 39 (53.4) | 99 (41.8) | .14 |
| Gynaecologist | 70 (95.9) | 198 (83.5) | **.001** |
| General practitioner | 35 (48) | 107 (45.2) | .61 |
| Midwife | 46 (63) | 137 (57.8) | .52 |
| Child & Family* | 25 (34.3) | 74 (31.2) | .31 |
| Prenatal sessions | 59 (80.8) | 77 (32.5) | **.04** |
| **INFORMATION TOPICS** |  |  |  |
| General health | 32 (43.8) | 119 (50.2) | .38 |
| Development of the baby | 66 (90.4) | 208 (87.78) | .78 |
| Health during pregnancy (lifestyle, nutrition, etc.) | 57 (78.1) | 191 (80.6) | .13 |
| Discomfort and complaints during pregnancy | 65 (89) | 188 (79.5) | **.02** |
| Labour and delivery | 42 (57.5) | 132 (55.7) | .65 |
| Postpartum | 33 (45.2) | 80 (33.8) | .15 |
| Breastfeeding | 42 (57.5) | 133 (56.1) | .67 |
| Bottle formula | 17 (23.3) | 40 (16.9) | .42 |
| Sexuality | 17 (23.3) | 45 (19) | .64 |
| Emotions and mental wellbeing | 24 (32.9) | 66 (27.9) | .21 |
| Administration and practical issues | 58 (79.5) | 175 (73.8) | .14 |
| **USE OF PREGNANCY APPS** |  |  |  |
| Did you use apps during your pregnancy? - Yes | 59 (80.8) | 181 (73.4) | .87 |
| *Child and Family (Kind en Gezin): aims to create as many opportunities as possible for all children and young people, including their families, who grow up in Flanders and Brussels. This Flemish service focuses on preventive treatment and guidance of young children geared to good outcomes in the future. They want to enable children to achieve their full developmental potential, physically, mentally, emotionally and socially, with respect for diversity and children’s rights.  NA = not applicable | | | |
